# Structural studies of haemoglobin from pisces species shortfin mako shark (*Isurus oxyrinchus)* at 1.9 Å resolution

**DOI:** 10.1107/S0909049513021572

**Published:** 2013-10-01

**Authors:** Pandian Ramesh, S. S. Sundaresan, Pon. Sathya Moorthy, M. Balasubramanian, M. N. Ponnuswamy

**Affiliations:** a Laboratory of Structural Biology, Department of Molecular Cell Biology, Samsung Biomedical Research Institute, Sungkyunkwan University School of Medicine, Suwon 440-746, South Korea; b Centre of Advanced Study in Crystallography and Biophysics, University of Madras, Maraimalai Campus (Guindy), Chennai 600 025, India

**Keywords:** haemoglobin, shark, monoclinic, oxygen transport, crystal structure, heme, tetramer

## Abstract

Haemoglobin (Hb) is a tetrameric iron-containing protein that carries oxygen from the lungs to tissues and carbon dioxide from tissues back to the lungs. Pisces are the advanced aquatic vertebrates capable of surviving at wide depth ranges. The shortfin mako shark (SMS) is the pelagic, largest, fastest and most sophisticated species of the shark kingdom with well developed eyes. Mostly the pisces species are cold blooded in nature. Distinctly, the SMSs are warm-blooded animals with an advanced circulatory system. SMSs are capable of maintaining elevated muscle temperatures up to 33 K above the ambient water temperatures at a depth of 150–500 m. SMSs have a diverged air-breathing mechanism compared with other vertebrates. The haemoglobin molecule consists of four polypeptide chains, namely two α chains, each with 140 amino acids and two β chains each having 136 amino acids. The SMS Hb was found to crystallize in monoclinic space group *P*2_1_ using the hanging-drop vapour-diffusion method at room temperature. The crystal packing parameters for the SMS Hb structure contain one whole biological molecule in the asymmetric unit with a solvent content of 47%. The SMS Hb quaternary structural features interface–interface interactions and heme binding sites are discussed with different state Hbs and the results reveal that SMS Hb adopts an unliganded deoxy T state conformation.

## Abstract 2

Shortfin mako shark haemoglobin adopts an unliganded deoxy T state conformation, which is shown from the quaternary structural features, interface interactions and heme binding sites of different subunits of haemoglobin with high-resolution X-ray data.

## 1. Introduction

Globins appear to have arisen very early in evolution and are found in organisms from bacteria to man. This selective success has been due to the functional flexibility of the protein, in particular, the ability of globins to interact with each other to give cooperative oxygen binding in a number of ways. Pisces are advanced aquatic vertebrates capable of surviving at wide depth ranges. The shortfin mako shark (SMS) is the pelagic, largest, fastest and most sophisticated species of the shark kingdom. Makos are well adapted with an extremely robust streamlined body with well developed eyes. Mostly the pisces species are cold-blooded in nature. Distinctly, the SMSs are warm-blooded animals with an advanced circulatory system which is capable of maintaining elevated muscle temperatures up to 33 K above the ambient water temperatures at a depth of 150–500 m with a diverged air-breathing mechanism compared with other vertebrates (Goodman *et al.*, 1987 ▶). The SMS haemoglobin (Hb) structure belongs to the family *Lamnidae* (Maia *et al.*, 2007 ▶). The α and β chains of the mustelus griseus and SMS Hb has 140 and 136 amino acids, respectively. The accession number for the amino acid sequence of the SMS is F2Z286 for α and F2Z287 for β chains. The sequence identity of SMS Hb compared with human and mustelus griseus shark Hb shares 41 and 80% for the α-chain and 35 and 66% for the β-chain, respectively (Naoi *et al.*, 2001 ▶). Even though a good amount of work has already been carried out using X-ray diffraction studies of Hbs from different species, there still exists a scarcity on structure–function information on sea-dwelling pisces species. An attempt has been made to gain a structure–function insight into the SMS (*Isurus oxyrinchus*) and its adaptation to the deep sea water environment by employing crystallographic methods.

## 2. Experiment

### 2.1. Purification and crystallization

Fresh whole blood was collected and subsequently mixed with 10% EDTA to avoid clotting. Red blood cells were isolated from the whole blood by centrifugation at 6000 r.p.m. for 20 min and the recovered precipitant was washed three times with two volumes of 0.9% (*w*/*v*) saline and haemolyzed by the addition of three times the volume of distilled water. The haemolyzed solution was centrifuged at 12000 r.p.m. for 1 h, which yielded cell-free Hb solution as the supernatant. The sample was applied onto an ion exchange chromatography column using DEAE-cellulose as the column material; the chromatographic column was equilibrated with water (Knapp *et al.*, 1999 ▶). The salt-gradient elution was achieved using a NaCl gradient from 0.1 to 1.0 *M*. The Hb was eluted at 0.1 *M* NaCl and was collected at a rate of 3 ml min^−1^. The purity of the SMS Hb is about 95% and yielded a single band on 12% native PAGE (Davis, 1964 ▶). Crystallization conditions of the Hb are screened under various concentrations of protein by varying precipitants from PEG 400–10000, changing the buffer and its pH varying from 6.8 to 8.3. Diffraction-quality crystals were obtained within a week by hanging-drop vapour-diffusion method from a drop containing 5 µl of protein solution with 2 µl of reservoir solution containing 35% PEG 3350 in millipore water equilibrated against 1 ml of reservoir solution.

### 2.2. Data collection and processing

A good quality crystal was mounted in a cryo-loop and treated with 20% glycerol solution prior to data collection to reduce the temperature damage. Intensity data were collected at 100 K on a rotating-anode Bruker Microstar X-ray source with a *mar345dtb* imaging-plate detector system. The SMS Hb crystal diffracted up to 1.9 Å resolution and data collection statistics are presented in Table 1 ▶. Collected data were indexed, integrated, merged and scaled using *AUTOMAR* and *SCALEPACK* software packages (Bartels & Klein, 2003 ▶). Evaluation of the crystal-packing parameters reveals that the lattice can accommodate one whole biological molecule in the asymmetric unit with a solvent content of 47.0% (Matthews, 1968 ▶).

### 2.3. Structure solution and refinement

The structure of Hb was solved by molecular-replacement method with MGS Hb as a starting model (PDB code 1gcv; Naoi *et al.*, 2001 ▶) using the program *PHASER* (McCoy *et al.*, 2007 ▶) and refinement carried out by *REFMAC 5.0* (Murshudov *et al.*, 1997 ▶) implemented in the CCP4 suite (Collaborative Computational Project, Number 4, 1994 ▶). The structure solution and refinement parameters are presented in Table 2 ▶. Further refinement was carried out using *REFMAC 5.0* and *PHENIX* (Afonine *et al.*, 2005 ▶). The final *R*/*R*_free_ values are 20.0%/25.7% for the data collected between 25.9 and 1.9 Å. The Ramachandran plot calculated for the final model with the programme *PROCHECK* (Laskowski *et al.*, 1993 ▶) shows 97.93 and 2.07% of the residues lie in the most favoured and allowed regions, respectively. The SMS Hb refined coordinates and structure factor files are deposited in the Protein Data Bank with the ID 3mkb.

## 3. Results and discussion

### 3.1. Structure of SMS Hb

**Figure 1:**
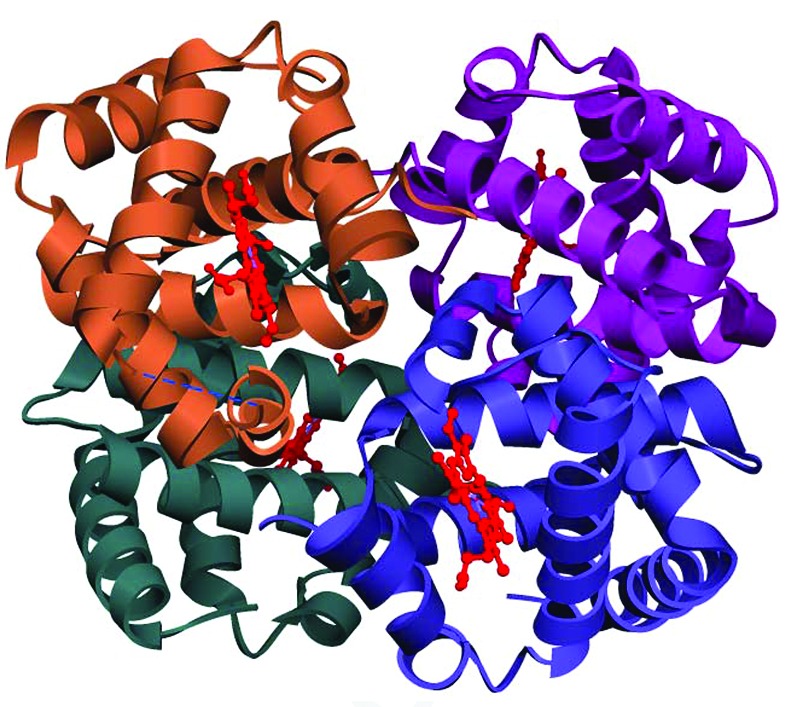
Crystal structure of SMS Hb in tetramer form with heme in each subunit.

The crystal structure of SMS Hb is presented in tetrameric form and each chain contains a heme group as shown in Fig. 1 ▶. The amino acid sequences of both α and β chains of SMS Hb are aligned in comparison with MGS Hb by using the *CLUSTALW2* program (Thompson *et al.*, 1997 ▶). The SMS α chain has 80% sequence identity whereas the β chain has only 68% with the MGS β chain. SMS and MGS have 140 residues in the α chain and 136 in the β chain. In the β chain, three deleted residues (45, 46 and 47) were found in between the C and E helices when compared with the sequence of MGS Hb. A similar kind of environment was also observed in the CO form of MGS Hb. All known cartilaginous fish Hb sequences have lost three and four residues between the C and E helices and are thought to lose the D helix in the β chain (Chong *et al.*, 1999 ▶). The highly conserved C-terminal sequences Tyr–Arg in the α chain and Tyr–His in the β chain are found in SMS and MGS Hbs.

**Figure 2:**
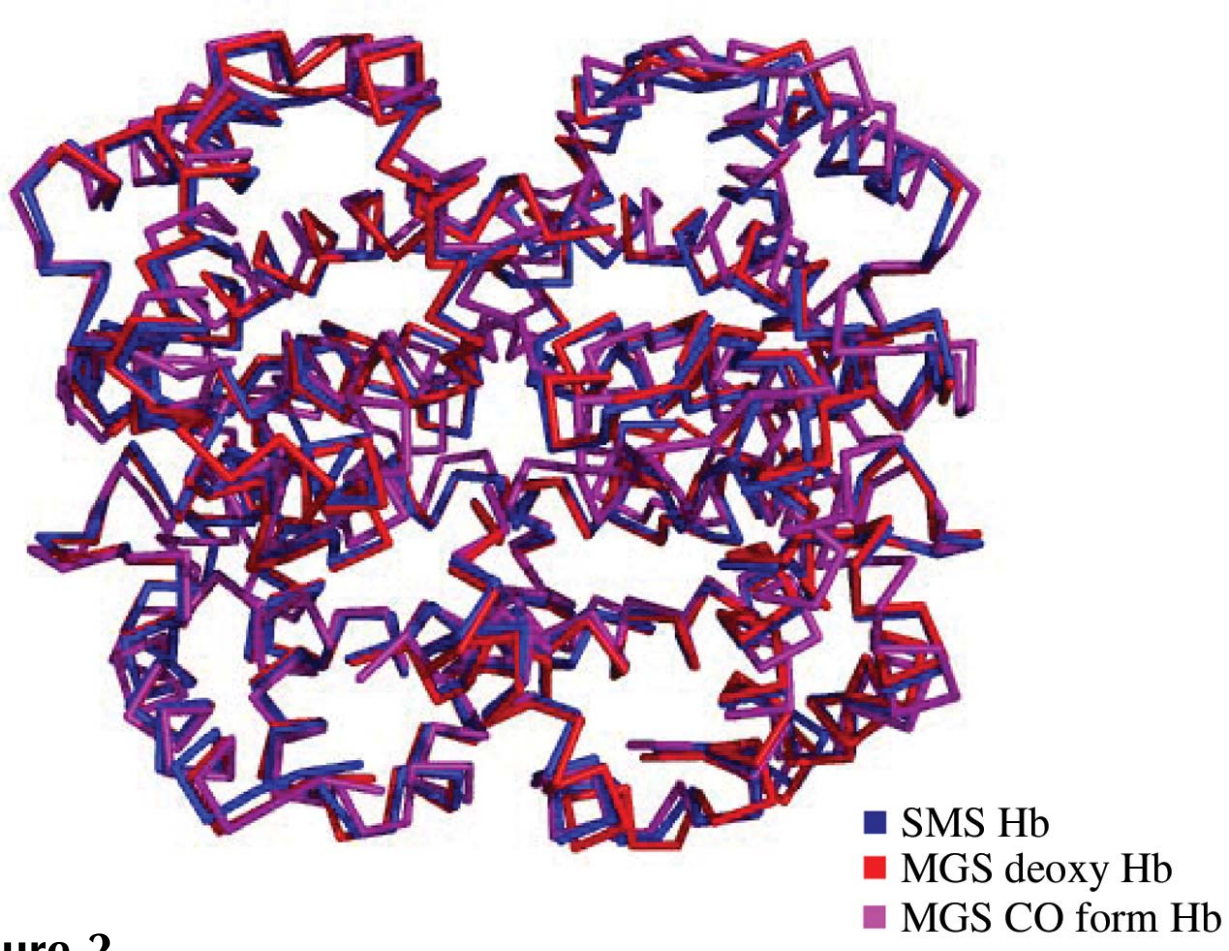
Superposition of α-traces SMS Hb with MGS deoxy and carbonmonoxy Hbs.

The difference in the quaternary structure between the tense (T) and the relaxed (R) states is primarily through a movement of the α2β2 dimer as a whole with respect to the α1β1 subunit. The deviations among the α1β1 subunit provide a measure of the differences in the structure of the dimer; those in α2β2 largely depend upon the differences in the mutual orientation of the two dimers. The superimposed root-mean-square deviation (RMSD) value between the MGS deoxy T state Hb and SMS Hb is 0.518 Å and between the MGS deoxy and MGS carbonmonoxy Hb is 0.912 Å, revealing a high degree of similarity in the α1β1 dimer. The RMSD value of the non-superimposed α2β2 dimer between SMS Hb and MGS deoxy T state Hb is 2.098 Å, which is smaller when compared with 11.170 Å for the MGS CO form R state. The rigid-body rotation angle between MGS deoxy T and SMS Hb is 0.854°, which is smaller compared with the other state Hb. The rotation angle between R state liganded MGS Hb and SMS Hb is 5.339°, which is comparable with the MGS deoxy T state value of 5.647°. The SMS Hb tetramer was superimposed with the unliganded deoxy (T) state and carbonmonoxy liganded states of MGS as shown in Fig. 2 ▶. The superimposed RMSD value between the SMS Hb and CO form of the MGS is 2.151 Å and with the unliganded state MGS Hb is 0.532 Å for all Cα atoms. From the quaternary structural features, it is learnt that the SMS Hb and MGS deoxy Hb quaternary structures are similar and adopt an unliganded deoxy T state.

### 3.2. Subunit interface interactions

**Figure 3:**
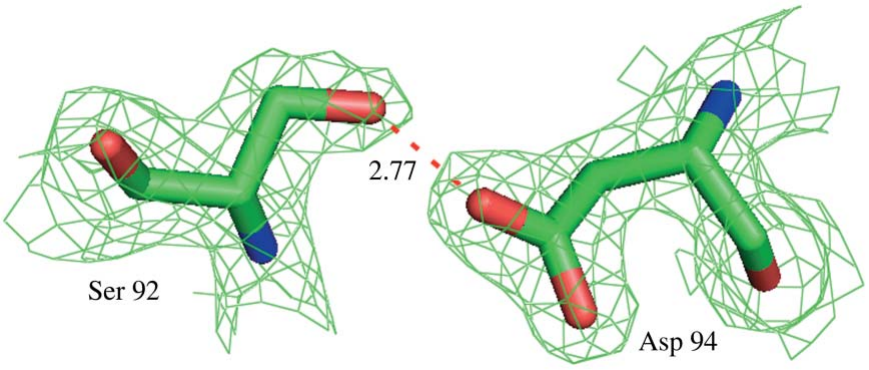
Electron density map of α1Asp94 and β2Ser92, contoured at the 1σ level. The dotted line in red shows the contact between α1Asp94 and β2Ser92.

The interface interactions play a major role in stabilizing the quaternary structure of Hb. An analysis of the contacts between the α1β1 interface of SMS Hb with the deoxy and CO form of MGS Hbs is shown in Table 3 ▶. The sequence identity between the MGS deoxy and SMS Hb in the α1β1 interface is ∼75%, slightly lower than the overall sequence value. However, the α1β1 interface region is highly conserved during evolution and this region is used as a reference frame in comparing structures, both liganded and unliganded forms (Ito *et al.*, 1995 ▶; Chong *et al.*, 1999 ▶; Baldwin & Chothia, 1979 ▶). The SMS Hb has four hydrogen bonds and 86 non-bonded contacts in the α1β1 interface region and shows the compact nature of the structure. Three hydrogen bonds out of four are strong when compared with the deoxy form of MGS Hb in the α1β1 interface. The different α1β2 contacts in SMS Hb show the loss of one hydrogen bond at position αSer96 due to the absence of a Gly carbonyl group. One notable hydrogen-bond interaction of the α1β2 interface region (α1Asp94–β2Ser92) is fairly strong at 2.77 Å compared with two other forms of deoxy and carbonmonoxy states MGS Hbs with varying lengths of 2.81 Å and 2.73 Å, respectively, as shown in Fig. 3 ▶. The total number of contacts in the α1β2 interface region reveals the changes from unliganded to liganded states of Hb, but in the α1β1 region the changes are minimum (Baldwin & Chothia, 1979 ▶). The polar contacts are defined as the interaction of 3.2 Å or less between polar and non-polar atoms. The interaction lengths of 3.2 Å to 3.5 Å in between polar atoms are counted as non-polar contacts (Mueser *et al.*, 2000 ▶). The total number of polar and non-polar contacts in the α1β2 interface region of T state Hb shows more contacts observed when compared with that of the R state. The α1β1 interface region of SMS Hb is more stable compared with both forms of MGS (deoxy and CO), owing to the presence of a number of contacts in the α1β2 interface region.

### 3.3. Heme binding site

**Figure 4:**
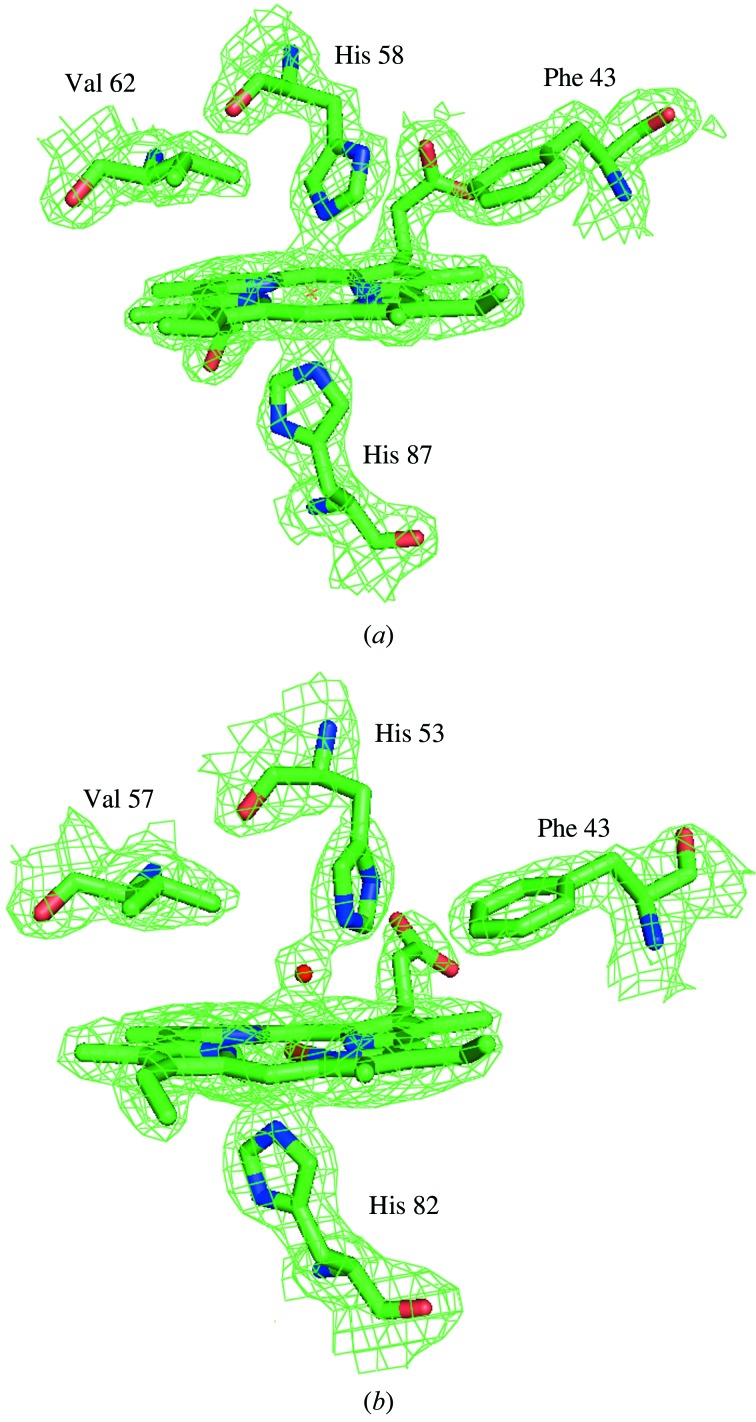
The 2*F*_o_ − *F*_c_ electron density map of the heme group region contoured at the 1.0σ level for (*a*) α1 and (*b*) β1 subunits of SMS Hb.

In SMS Hb, β heme groups and the coordinated water O atoms are well defined in the 2*F*_o_ − *F*_c_ electron density map, whereas α heme groups do not contain the water molecule and their surrounding environment is shown in Fig. 4 ▶. A significant difference noted between SMS and MGS deoxy Hb is the presence of a distal water molecule in the β heme pocket of the SMS Hb (Fig. 4*b* ▶). The heme pocket water is hydrogen-bonded to N^∊^ of His-53 in many of the deoxy β subunit Hb structures. This water molecule has to be displaced before ligation so that the ligand movement into the distal pocket is greatly inhibited. The stereochemistry of the heme group and the surrounding environment in SMS Hb in comparison with other pisces are presented in Table 4 ▶. Another notable difference is the position of βVal67 in human Hb which lies close (3.94 Å) to the Fe atom and weakens the ligand binding in the T state (Fermi *et al.*, 1984 ▶). The residue βVal67 in fish *D. akajei* Hb is relatively far from the ligand binding site (Chong *et al.*, 1999 ▶), suggesting the possibility of a high oxygen affinity. This residue is located far away from the iron atom compared with human and suggests the possibility of a high oxygen affinity in MGS and SMS Hbs. During the transition from the unliganded to the liganded state, some difference in the heme–heme distance and change of geometry are noted in different state Hb structures. In the MGS T structure the total Fe—Fe distance is 133.77 Å, followed by that observed in the R3 state (126.92 Å). In the T and R3 structures the β1Fe—β2Fe observed distance has the longest and shortest value, respectively. The different liganded structures may exhibit different affinities for oxygen, depending on the size of the β-cleft (Safo & Abraham, 2005 ▶). The total iron–iron distance of SMS Hb, 135.35 Å, is comparable with the T state, while in MGS Hb the total iron–iron distance difference is more when compared with other liganded Hbs. The α and β chain heme groups of SMS compared with the unliganded state of MGS deoxy and carbonmonoxy Hb show RMSD values of 0.449 Å and 0.675 Å for α and 0.355 Å and 0.877 Å for β subunits, respectively.

## 4. Conclusions

Shortfin mako shark is a salt-water fish belonging to the pisces family and crystallizes in monoclinic space group *P*2_1_. SMS has 140 and 136 residues in the α and β chains. In the β chain, three deleted residues (45, 46 and 47) were found between the C and E helices when compared with the three-dimensional structure of MGS, and similar kinds of missing residues are also observed in the CO form of MGS Hb. The SMS has 80% and 68% sequence similarity with the α and β chains of MGS. The SMS Hb has a higher number of hydrogen bonds and non-bonded contacts in the α1β1 interface region, which shows the stability of the structure. The study reveals that SMS Hb adopts an unliganded deoxy T state conformation, which is shown from the results of quaternary structural features, interface interactions and heme binding sites of different states Hbs.

## Supplementary Material

PDB reference: 3mkb

## Figures and Tables

**Table 1:** Data collection statistics for SMS Hb Values in parentheses are for the highest-resolution shell.

X-ray source/wavelength (Å)	Cu *K*α/1.5418
Temperature (K)	100
Oscillation angle (°)	1
Exposure time (min)	1
Space group	*P*2_1_
Crystal size (mm)	0.35 × 0.27 × 0.20
Crystal to detector distance (mm)	150
Unit-cell parameters (Å, °)	*a* = 60.259, *b* = 61.099, *c* = 72.207, β = 96.49
Resolution range (Å)	25.9–1.9 (1.97–1.9)
Observed/unique reflections	117478/41106
Matthew coefficient, *V*_M_ (Å^3^ Da^−1^)	2.19
Solvent content (%)	47%
No. molecules in atomic unit	1
*R*_merge_ (%)	7.56 (37.45)
Average redundancy	2.79 (2.78)
Completeness	99.2 (98.8)
Average *I*/σ (*I*)	4.7 (0.9)

**Table 2:** Structure solution and refinement statistics for SMS Hb

Structure solution and refinement	
Resolution range (Å)	25.9–1.9
Reflections used	40915
Initial *R*-factor/*R*_free_ (%)	42.7/44.1
Final *R*-factor/*R*_free_ (%)	20.0/25.7
R.m.s deviations from ideals	
Bond length (Å)	0.009
Bond angle (°)	1.060
Chiral volume (Å^3^)	0.069
Mean *B* values (Å^2^)	26.89
Ramachandran plot	
Residues in most favourable region (%)	97.93
Residues in allowed region (%)	2.07

**Table 3:** The α1β1 interface interactions (Å) of pisces Hbs Amino acid replacement of SMS Hb shown in parentheses.

Residues	SMS Hb	MGS deoxy Hb	MGS CO Hb
Arg31–Phe112	2.92	3.02	3.19
Arg31–Gln117(Phe)	3.37	3.04	3.02
Arg31–Phe112	2.82	2.84	2.81
(Ala)Thr114–Tyr106(His)	4.75†	2.29	2.51
Phe116–Arg30	2.96	3.06	3.12
Phe116–Arg30	2.96	2.63	2.71
(Ala)Asp119–Lys33(Val)	3.95†	3.12	3.15
His121–Arg30	3.12	3.01	3.17

† Minimum distance between two residues.

**Table 4:** Geometry of heme groups and environment of pisces Hbs in different forms (Å)

	SMS Hb		MGS deoxy Hb		MGS CO Hb	
	α1	β1	α1	β1	α1	β1
Fe-His (E7) E2	2.48	3.87	4.20	4.29	5.62	4.74
Fe-Val (E11) CG2	4.74	4.68	4.77	4.96	5.13	5.24
Fe-Phe (CD1) CZ	6.04	6.06	6.41	6.25	5.52	5.22
Fe-His (F8) NE2	2.31	2.42	2.18	2.24	2.22	2.26
